# The Role of Omentin in Gastrointestinal Cancer: Diagnostic, Prognostic, and Therapeutic Perspectives

**DOI:** 10.3390/metabo15100649

**Published:** 2025-09-30

**Authors:** Adam Mylonakis, Maximos Frountzas, Irene Lidoriki, Alexandros Kozadinos, Maria Evangelia Koloutsou, Angeliki Margoni, Areti Kalfoutzou, Dimitrios Theodorou, Konstantinos G. Toutouzas, Dimitrios Schizas

**Affiliations:** 1 First Department of Surgery, National and Kapodistrian University of Athens, Laikon General Hospital, 11527 Athens, Greece; adam.mylonakis@gmail.com (A.M.); alexkozadinos@gmail.com (A.K.); schizasad@gmail.com (D.S.); 2 Department of Colorectal Surgery, Royal Marsden NHS Foundation Trust, London SWE 6JJ, UK; froumax@med.uoa.gr; 3 Department of Environmental, Occupational Medicine and Epidemiology, Harvard T.H. Chan School of Public Health, Boston, MA 02115, USA; 4 First Department of Propaedeutic Internal Medicine, National and Kapodistrian University of Athens, Laikon General Hospital, 11527 Athens, Greece; obbonesphd@gmail.com; 5 Department of Biological Chemistry, Medical School, National and Kapodistrian University of Athens, 11527 Athens, Greece; angeliki.margoni@gmail.com; 6 Oncology Unit, Second Department of Internal Medicine, National and Kapodistrian University of Athens, Attikon University Hospital, 12462 Athens, Greece; 7 First Department of Propaedeutic Surgery, National and Kapodistrian University of Athens, Hippokrateion General Hospital, 11527 Athens, Greece; dimitheod@netscape.net (D.T.); tousur@med.uoa.gr (K.G.T.)

**Keywords:** omentin, gastrointestinal neoplasms, adipokines, tumor microenvironment, biomarker

## Abstract

**Background/Objectives**: Omentin, also known as intelectin-1, is a secreted adipokine with anti-inflammatory, insulin-sensitizing, and immune-modulatory functions, primarily expressed in visceral adipose tissue. While omentin has been associated with favorable metabolic outcomes, its role in cancer pathogenesis appears context-dependent and remains poorly understood. This review investigates the biological functions, expression patterns, and clinical relevance of omentin across gastrointestinal malignancies. **Methods**: A comprehensive review of the literature was conducted using PubMed, Scopus, and Web of Science up to August 2025 to evaluate the role of omentin in gastrointestinal cancers. Both preclinical and clinical studies evaluating omentin, its analogues and omentin-enhancing agents in gastric, colorectal, hepatic, pancreatic, and esophageal cancers were included. **Results**: Omentin exhibits anti-proliferative, anti-inflammatory, and anti-angiogenic effects within the tumor microenvironment in several GI malignancies. However, evidence also indicates a dual role. High intratumoral omentin expression correlates with improved prognosis in colorectal, gastric, and hepatic cancers; in contrast, elevated circulating levels–particularly in colorectal and pancreatic cancers–have been paradoxically associated with increased cancer risk and poor outcomes. Mechanistically, omentin modulates PI3K/Akt, NF-κB, AMPK, and oxidative stress pathways, and interacts with TMEM207. However, most available studies are small-scale and heterogeneous, with methodological inconsistencies and limited multi-omics integration, leaving major knowledge gaps. **Conclusions**: This review highlights omentin’s distinct systemic and local roles across GI cancers, underscoring its translational implications. Omentin emerges as a promising but context-dependent biomarker and therapeutic target, with future research needed to address heterogeneity, standardize assays, and validate its clinical utility in large-scale prospective studies.

## 1. Introduction

Gastrointestinal (GI) cancers remain among the leading causes of cancer-related morbidity and mortality worldwide. Despite advances in surgical technique and medical oncology, outcomes for many patients with GI cancers—especially at advanced stages—remain poor [1]. This underscores the need for novel biomarkers that could aid in early detection, risk stratification, and therapeutic guidance.

Omentin (also Omentin-1, intelectin-1, ITLN1) is an adipokine primarily produced in visceral adipose tissue, particularly within omental fat depots. In contrast to many adipokines that are pro-inflammatory, omentin is generally considered a beneficial adipose-derived factor with metabolic and anti-inflammatory roles [2]. Biologically, omentin-1 exerts insulin-sensitizing effects—enhancing insulin-mediated glucose uptake in adipocytes—and displays broad anti-inflammatory and anti-atherogenic functions [3]. Notably, circulating omentin-1 levels are inversely associated with obesity and related metabolic disorders. Omentin expression is reduced in conditions of visceral obesity, insulin resistance, and type 2 diabetes, whereas weight loss or exercise can raise omentin-1 circulating levels [4,5,6,7,8].

The observation that pro-tumor metabolic conditions coincide with low omentin has prompted interest in the possible influence of this adipokine on cancer development. It is hypothesized that dysregulation of adipokines, such as omentin, could explain why metabolic syndrome contributes to tumorigenesis in organs like the stomach, colon, and esophagus [9]. These discrepancies underscore the dual, context-dependent role of omentin and the importance of distinguishing between systemic and local effects.

Emerging evidence suggests that omentin plays a role in cancer biology. In gastrointestinal cancers, intelectin-1 gene expression has been reported to be higher in tumor tissues compared to adjacent normal tissues. Patients with GI tumors also often exhibit elevated circulating omentin-1 levels compared to healthy controls [10]. Despite growing interest, significant gaps remain regarding the precise role of omentin-1 in the initiation and progression of GI malignancies. The prognostic and diagnostic value of circulating omentin-1 remains uncertain due to heterogeneity in existing studies’ design, small sample sizes, and variations in detection methods. Furthermore, the molecular mechanisms of interaction between omentin-1 and cancer-related pathways are only partially understood, and its potential as a therapeutic target has yet to be thoroughly investigated.

To date, no comprehensive review has synthesized omentin’s systemic versus tumor-local roles across different GI malignancies. This review aims to synthesize current evidence on the biological role and clinical relevance of omentin in gastrointestinal cancers. By highlighting emerging findings and key areas of uncertainty, we seek to suggest future research directions and explore the potential for omentin as a novel biomarker and therapeutic target in GI oncology.

## 2. Methods

This review was conducted according to the principles of the Preferred Reporting Items for Systematic Reviews and Meta-Analyses (PRISMA) guidelines, adapted for a narrative review [11]. A comprehensive literature search was performed in PubMed/MEDLINE, Scopus, and Web of Science for articles published up to August 2025. The following search terms were used in various combinations: “omentin” OR “omentin-1” OR “intelectin-1” OR “ITLN1” AND “cancer” OR “neoplasm” OR “malignancy” OR “tumor” AND “gastrointestinal” OR “gastric” OR “colorectal” OR “esophageal” OR “pancreatic” OR “hepatic”.

Our review included 21 studies assessing the role of omentin in gastrointestinal cancers: 15 clinical and 6 preclinical/mechanistic. A flow chart of the study selection process is provided in Supplementary Figure S1.

## 3. Protective Biological Functions of Omentin

Omentin (intelectin-1) is a secreted glycoprotein adipokine encoded by the ITLN1 gene and predominantly produced in visceral adipose tissue [12]. Humans express two omentin isoforms, omentin-1 and omentin-2, which share ~83% amino acid identity and are encoded by adjacent genes (ITLN1 and ITLN2) clustered on chromosome 1q22–q23 [13].

Omentin-1 is highly expressed in visceral fat depots (especially omental and epicardial fat) and is secreted into circulation, whereas expression in subcutaneous fat is much lower [14]. Notably, omentin is mainly produced by stromal-vascular cells within adipose tissue rather than by adipocytes themselves [15]. Beyond adipose tissue, omentin is found in multiple tissues and cell types: it is expressed by mesothelial cells (e.g., in the peritoneum), endothelial and other vascular cells, and prominently by certain gut epithelial cells [15]. In the digestive tract, high omentin levels are seen in goblet and Paneth cells of the small intestine and colon, from which it is secreted into the mucus [16]. Omentin is also detected in the respiratory tract (airway goblet cells and glands), ovaries, placenta, and other tissues [17,18,19]. This wide distribution, particularly at barrier surfaces, suggests a role in immune surveillance in addition to its endocrine function as an adipokine [17,20,21].

Of the two isoforms, omentin-1 is the predominant circulating and tissue form studied in humans, whereas omentin-2 is expressed at much lower levels and its biological significance remains less defined. Current evidence suggests that isoform differences are mainly relevant in the context of metabolic regulation, while almost all cancer-related studies have focused exclusively on omentin-1 [13,18]. Thus, the role of omentin-2 in gastrointestinal malignancies remains uncertain.

### 3.1. Anti-Inflammatory and Immune Regulatory Roles

Omentin-1 exerts notable anti-inflammatory effects in various cell types and tissues. It generally acts to dampen pro-inflammatory signaling pathways and cytokine production. For example, omentin-1 inhibits the expression of adhesion molecules (VCAM-1, ICAM-1) and suppresses inflammatory cytokine release in endothelial cells by blocking key stress-activated pathways (p38 MAPK, c-Jun N-terminal kinase- JNK) and the Nuclear factor kappa-light-chain-enhancer of activated B cells (NF-κB) transcription factor [13]. In human endothelial and adipose cells exposed to inflammatory stimuli (like free fatty acids or TNF-α), omentin-1 reduces NF-κB activation and lowers production of cytokines such as TNF-α, IL-6, IL-1β, and chemokine MCP-1 [22,23]. Several studies confirm that omentin’s anti-inflammatory action involves intracellular signaling modulation—for instance, activating AMP-activated protein kinase (AMPK) and Protein kinase B (Akt), while antagonizing pro-inflammatory kinases [15,24]. In obese mice, omentin derived from perivascular fat was found to increase anti-inflammatory mediators (interleukin-10 and adiponectin) and concurrently decrease levels of TNF-α, IL-6, and IL-1β in tissues by inhibiting the TXNIP/NLRP3 inflammasome pathway [25].

In addition to blunting inflammatory signaling, omentin plays a direct role in immune regulation and host defense. As a lectin, omentin can recognize and bind to carbohydrate antigens present on microbial pathogens, thereby functioning in innate immunity. It selectively binds microbial exocyclic 1,2-diol motifs (such as β-D-galactofuranose found in bacteria) and can agglutinate bacteria upon binding [21]. Omentin-1 is present at high concentration in the intestinal mucus and on mesothelial surfaces, which suggests it contributes to the first-line immune defense by blocking pathogen entry and facilitating neutrophil/macrophage uptake of invaders [25]. Furthermore, omentin-1 can modulate immune cell function; treating macrophages with recombinant omentin-1 in vitro was shown to attenuate lipopolysaccharide-induced expression of pro-inflammatory genes and to reduce foam cell formation (lipid accumulation) in these cells [25]. These effects were associated with activation of the Akt signaling pathway in macrophages, and pharmacologic inhibition of Akt abolished omentin’s anti-inflammatory influence [25]. Taken together, such findings indicate that omentin not only serves as an opsonin-like innate immune lectin but also actively regulates inflammatory cell signaling, biasing immune responses toward an anti-inflammatory, tissue-protective state.

### 3.2. Antioxidant Effects and Oxidative Stress Modulation

Another important function of omentin is the mitigation of oxidative stress in tissues. Chronic inflammation often coincides with elevated reactive oxygen species (ROS) and oxidative damage, and omentin-1 appears to counteract these processes. In vascular cells, omentin has demonstrated antioxidant effects: for example, it can inhibit NADPH oxidase-mediated superoxide production triggered by TNF-α in vascular smooth muscle cells, thereby reducing oxidative stress and inflammation in the vascular wall [26,27]. Omentin-1 also protects the endoplasmic reticulum and endothelium from oxidative injury by enhancing nitric oxide (NO) bioavailability, partly through activating peroxisome proliferator-activated receptor-δ (PPAR-δ) and AMPK, which in turn upregulates endothelial NO synthase and antioxidant defenses [28]. In models of acute inflammation, omentin was shown to activate the Nrf2 signaling pathway, a master regulator of cellular antioxidant responses [29].

Direct evidence of omentin’s cytoprotective antioxidant action comes from endothelial cell experiments. Human endothelial cells pretreated with omentin-1 are significantly more resistant to oxidative injury. In-vitro assays showed that omentin-1 prevented hydrogen peroxide (H_2_O_2_)-induced apoptosis of endothelial cells and reduced intracellular ROS accumulation. Such findings underscore that omentin-1 serves as an antioxidant adipokine: by lowering oxidative stress and enhancing antioxidant enzymes, it helps shield cells (endothelium, immune cells, etc.) from oxidative damage. These antioxidant properties of omentin likely contribute to its anti-inflammatory and vascular-protective effects, since reduced oxidative stress limits the activation of inflammatory pathways and endothelial dysfunction [30].

### 3.3. Vascular Biology and Vascular Protective Functions

Omentin-1 plays an important role in vascular function, generally exerting vasoprotective and anti-atherogenic effects. One key function is promoting healthy endothelial function and vasodilation. Omentin-1 stimulates the production of nitric oxide (NO) in endothelial cells, leading to endothelium-dependent relaxation of blood vessels, by activating endothelial NO synthase (eNOS) via Akt/AMPK signaling, thereby increasing NO release and supporting vascular homeostasis [15].

Omentin’s anti-inflammatory properties are particularly beneficial in the vasculature, where chronic inflammation drives atherosclerosis and vascular damage. Omentin-1 suppresses endothelial activation by reducing expression of adhesion molecules (ICAM-1, VCAM-1) that recruit leukocytes to the vessel wall [26,31,32]. By inhibiting NF-κB and MAPK signaling in vascular cells, omentin curtails the cascade of inflammation that leads to endothelial dysfunction and plaque formation [22]. The adipokine is therefore considered anti-atherogenic. Indeed, in vivo, omentin slows atherosclerosis: in apolipoprotein E–deficient mice, transgenic overexpression or chronic infusion reduced aortic plaque size, macrophage infiltration, and arterial levels of proinflammatory mediators such as TNF-α, IL-6, and MCP-1 [24,33]. In vitro, omentin-1 reduced oxidized LDL uptake and foam-cell formation in macrophages, suppressed LPS-induced cytokine release, and promoted a less inflammatory, cholesterol-effluxing phenotype via Akt signaling [24,33]. Beyond inhibiting atheroma progression, omentin enhances angiogenesis and protects against ischemia–reperfusion injury by activating PI3K/Akt and reducing oxidative stress in endothelial cells [33]. Clinically, higher circulating omentin correlates with better cardiovascular outcomes, whereas obesity and diabetes lower levels and contribute to endothelial dysfunction [34].

**Figure 1:**
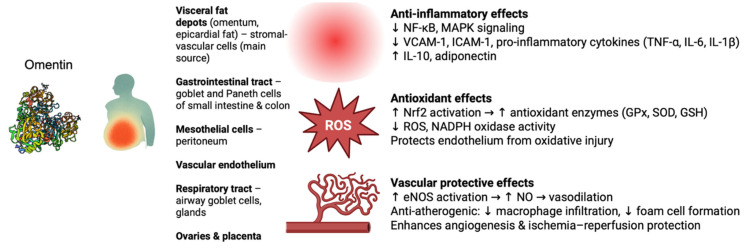
Overview of omentin expression and protective functions. Omentin, mainly secreted by visceral fat depots and expressed in gastrointestinal, mesothelial, vascular, respiratory, and reproductive tissues, exerts multiple protective effects. It suppresses inflammation (↓ NF-κB/MAPK signaling, ↓ VCAM-1/ICAM-1, ↓ TNF-α/IL-6/IL-1β, ↑ IL-10, adiponectin), reduces oxidative stress (↑ Nrf2-driven antioxidant enzymes, ↓ ROS, NADPH oxidase activity), and protects the vasculature (↑ eNOS–NO–mediated vasodilation, anti-atherogenic activity, enhanced angiogenesis and ischemia–reperfusion protection). NF-κB, nuclear factor kappa B; MAPK, mitogen-activated protein kinase; VCAM-1, vascular cell adhesion molecule-1; ICAM-1, intercellular adhesion molecule-1; TNF-α, tumor necrosis factor alpha; IL-6, interleukin-6; IL-1β, interleukin-1 beta; IL-10, interleukin-10; Nrf2, nuclear factor erythroid 2–related factor 2; GPx, glutathione peroxidase; SOD, superoxide dismutase; GSH, glutathione; ROS, reactive oxygen species; NADPH, nicotinamide adenine dinucleotide phosphate; eNOS, endothelial nitric oxide synthase; NO, nitric oxide.

In summary, omentin serves as an anti-inflammatory, antioxidant guardian of the vasculature: it promotes endothelial-dependent vasodilation, inhibits inflammatory adhesion and infiltration in vessels, and prevents the development of atherosclerotic changes as presented in Figure 1 These properties make omentin-1 an adipokine with significant cardiovascular protective potential independent of its metabolic effects.

## 4. The Role of Omentin in Metabolic Disorders

Metabolic disorders such as obesity, type 2 diabetes mellitus, metabolic syndrome, and Metabolic-associated steatotic liver disease (MASLD) are increasingly recognized as major risk factors for the initiation and progression of gastrointestinal malignancies. Chronic low-grade inflammation, hyperinsulinemia, insulin resistance, and dysregulated adipokine secretion create a systemic pro-tumorigenic environment [1,9]. These alterations promote DNA damage, oxidative stress, and angiogenesis while impairing immune surveillance, thereby facilitating tumorigenesis in organs such as the stomach, colon, liver, and pancreas [35,36].

### 4.1. Omentin and Obesity

Omentin-1 was first identified in stromal vascular cells of the visceral adipose tissue, and its levels have a notable inverse relationship with obesity. Numerous studies report that circulating omentin-1 is negatively correlated with body mass index (BMI), waist circumference, and adiposity in both adults and children [4,5,6]. In fact, weight reduction through diet or exercise have been shown to increase circulating omentin-1 concentrations [7,8]. This paradox reflects the fact that omentin is secreted mainly by stromal vascular cells rather than adipocytes, and its expression is downregulated by the chronic low-grade inflammation characteristic of obesity [8,9,10].

### 4.2. Omentin, Insulin Resistance and Diabetes

Omentin is a key adipokine involved in glucose metabolism and insulin sensitivity, with accumulating evidence suggesting that its reduced levels contribute to the pathophysiology of type 2 diabetes mellitus (T2DM). In vitro studies have shown that omentin enhances insulin signal transduction by activating protein kinase B (Akt), thereby improving the insulin responsiveness of adipose tissue [12]. Therefore, a lower circulating concentration of omentin might play a mechanistic role in the development of insulin resistance [4]. Clinically, decreased plasma omentin levels have been consistently observed in patients with T2DM, while individual studies have also assessed parameters such as fasting glucose and the Homeostatic model assessment of insulin resistance (HOMA-IR) index [4,37]. Notably, the reduction in omentin is not limited to overweight individuals; patients with normal BMI and T2DM also display significantly lower omentin concentrations than healthy controls, indicating that the reduction is not solely attributable to obesity [4]. Furthermore, reduced omentin levels have been linked to diabetes-related microvascular complications; patients with diabetic retinopathy, neuropathy, or nephropathy consistently show significantly lower circulating omentin compared with those without such complications, partly through mechanisms involving downregulation of vascular endothelial growth factor (VEGF). Interestingly, while the relationship between omentin and T2DM is well established, findings in type 1 diabetes mellitus (T1DM) remain inconsistent. Some studies report decreased plasma omentin levels in T1DM, whereas others have observed elevated concentrations, suggesting a divergent regulatory mechanism or role for omentin in this context [37].

### 4.3. Omentin and Metabolic Syndrome

Metabolic syndrome is a cluster of interconnected risk factors—including abdominal obesity, dyslipidemia, hypertension, and hyperglycemia—that predispose individuals to type 2 diabetes and cardiovascular disease. Omentin-1 appears to be closely associated with these risk factors. As previously discussed, reduced omentin-1 levels are commonly observed in conditions such as obesity, insulin resistance, and type 2 diabetes—all key components of metabolic syndrome [4,12,18]. Furthermore, omentin-1 is positively correlated with plasma levels of high-density lipoprotein (HDL) and negatively with very low-density lipoprotein (VLDL), which is a lipoprotein linked to atherogenic lipid profiles [5]. Studies investigating metabolic syndrome have consistently found that affected individuals tend to have lower circulating omentin-1 levels compared to healthy controls, a finding that aligns with omentin’s inverse association with body mass index (BMI), triglyceride levels, and insulin resistance [38,39,40]. Notably, there is also evidence that omentin-1 may have predictive value. In a prospective study of individuals with hypertension and increased risk for metabolic syndrome, lower baseline omentin-1 concentrations were associated with a higher likelihood of developing the full syndrome during follow-up [39]. Collectively, these findings suggest that omentin-1 is not only a sensitive marker of existing metabolic dysregulation, but it might also have predictive value for identifying individuals at risk of progressing to overt metabolic syndrome.

### 4.4. Omentin and Metabolic–Associated Steatotic Liver Disease

Metabolic-associated steatotic liver disease is associated with dysregulation of omentin-1, an insulin-sensitizing and anti-inflammatory adipokine. Clinically, patients with Metabolic dysfunction-associated steatotic liver disease (MASLD) consistently exhibit significantly lower circulating omentin-1 levels compared to healthy controls [35]. Moreover, in obese individuals, those who progress to steatohepatitis (MASH) usually show a further reduction in omentin-1 levels (independent of fibrosis), suggesting an inverse relationship between omentin and disease severity. Diminished omentin-1 might exacerbate MASLD by worsening insulin resistance and inflammation: low omentin correlates with higher glycemia and HOMA-IR, whereas normal omentin enhances insulin-stimulated glucose uptake via Akt and suppresses macrophage NF-κB activation, thereby mitigating systemic insulin resistance and inflammatory signaling [12,35]. In vitro and animal studies support a protective role for omentin-1 in the liver. Administration of omentin-1 in high-fat diet mouse models reduces hepatic fat accumulation and improves metabolic homeostasis, largely by activating AMPK and restoring autophagy in hepatocytes [41]. Similarly, treating fat-laden hepatocyte cultures with omentin-1 reduced TNF-α levels, ER stress, and oxidative stress via inhibition of NF-κB/MAPK signaling [36]. Through these actions, omentin-1 appears to counteract the key pathophysiological drivers of MASLD—hepatic steatosis, insulin resistance, and chronic inflammation—potentially slowing progression to fibrosis. Consistently, experimental models have reported that omentin-1 therapy can ameliorate steatosis and prevent fibrotic changes [36,41]. In summary, omentin plays a protective role in MASLD pathogenesis and confers metabolic and anti-inflammatory benefits that are diagnostically and therapeutically relevant in the context of fatty liver disease.

**Figure 2:**
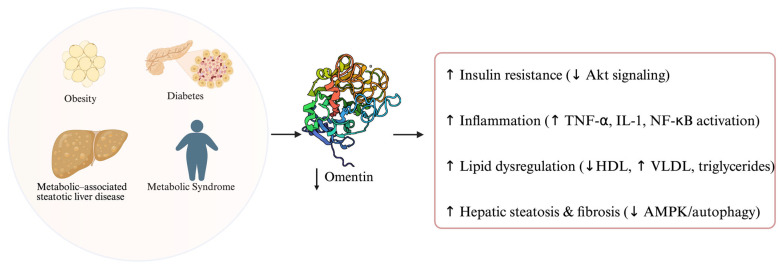
Schematic representation of the role of omentin in metabolic disorders. Akt, protein kinase B; TNF-α, tumor necrosis factor alpha; IL-1, interleukin-1; NF-κB, nuclear factor kappa B; HDL, high-density lipoprotein; VLDL, very low-density lipoprotein; AMPK, AMP-activated protein kinase.

An overview of the role of omentin in metabolic disorders is presented in Figure 2.

## 5. The Role of Omentin in Gastrointestinal Cancers

### 5.1. Upper Gastrointestinal Cancer

In their cross-sectional analysis of adipose tissue from upper-gastrointestinal cancer patients, Miller et al. found that omentin was the single most increased transcript in visceral adipose tissue (VAT). Compared with healthy kidney-donor controls, omentin mRNA had risen nine-fold in weight-stable cancer patients and ten-fold in cachectic patients, while remaining unchanged in subcutaneous fat. Gene-set enrichment showed that this sharp induction occurred against a background of suppressed adipogenesis and oxidative-phosphorylation programs, suggesting that VAT responds to tumor burden by shifting away from energy-storage genes and strongly favoring omentin expression. Protein assays confirmed the transcriptional signal. Omentin concentrations in VAT were lowest in controls (≈20 ng mL^−1^), intermediate in weight-stable cancer (≈31 ng mL^−1^), and highest in cachectic patients (≈44 ng mL^−1^), indicating a disease- and weight-loss-dependent escalation. Circulating omentin showed greater inter-individual variability, but cachectic patients still displayed significantly higher plasma levels than weight-stable counterparts. These findings suggested that omentin is selectively amplified in gastrointestinal cancer—particularly when cachexia is present—implicating it as a candidate mediator of fat–muscle cross-talk and a potential therapeutic target in cancer-associated metabolic dysfunction [42].

#### 5.1.1. Esophageal Cancer

Esophageal cancer, including squamous cell carcinoma (ESCC) and adenocarcinoma (EAC), has not been studied as extensively as other GI cancers in the context of omentin. Esophageal adenocarcinoma shares risk factors with gastric cancer (notably obesity and gastroesophageal reflux leading to intestinal metaplasia), which suggests adipokines might be relevant [43]. However, specific data on omentin-1 in esophageal cancer are limited. To date, no large clinical studies were identified that directly measure omentin levels in esophageal cancer patients versus controls.

In a multi-gene prognostic model developed for ESCC, ITLN1 emerged as one of only two protective genes contributing to a composite immune-related risk signature. Higher ITLN1 expression was associated with lower calculated risk scores and independently predicted both improved overall and recurrence-free survival across two cohorts, one based on microarray data (GSE53624) and another validated by Quantitative real-time polymerase chain reaction (qRT-PCR). Although functional experiments were not performed, the authors proposed that elevated omentin-1 may reflect a more immunocompetent tumor microenvironment, consistent with prior reports of its tumor-suppressive role in gastric and colorectal cancers [44].

Complementing these findings, a separate glycolipid metabolism-focused transcriptomic analysis in ESCC also identified ITLN1 as a protective factor. The gene was downregulated in tumor tissue compared to adjacent normal mucosa, and its expression inversely correlated with poor prognostic features. Although no functional experiments were conducted on ITLN1, the authors noted its known anti-inflammatory actions—particularly its ability to dampen macrophage NF-κB signaling—which fits the study’s broader finding that high-risk ESCC tumors contain fewer B-cells and NK-cells and a more immunosuppressive micro- environment. They suggested that loss of omentin-1 is part of a metabolic and immune shift toward poorer prognosis, whereas retaining or restoring omentin-1 activity might help maintain anti-tumor immunity in ESCC [45].

In contrast, studies of esophageal adenocarcinoma have positioned ITLN1 not as a dynamic biomarker of progression, but rather as a stable marker of Barrett’s esophagus (BE), the metaplastic precursor lesion. A single-cell RNA-sequencing study demonstrated that ITLN1 is consistently enriched in goblet-cell–like populations within BE but does not change meaningfully across progression stages (non-dysplastic BE, low- and high-grade dysplasia, and focal adenocarcinoma). In short, the paper confirmed that omentin-1 is a characteristic transcript of Barrett’s metaplasia, but it provided no evidence that changes in its level signal or drive predict the transition to esophageal adenocarcinoma [46].

#### 5.1.2. Gastric Cancer

The role of omentin-1 in gastric cancer has been explored both at the tissue and mechanistic levels, with consistent evidence pointing toward a tumor-suppressive function. In a single-center study of 196 gastric adenocarcinoma patients, Zheng et al. demonstrated an approximately eight-fold increase in ITLN1 mRNA in tumors compared to paired normal mucosa, confirmed by qPCR (*p* < 0.001). Immunohistochemical analysis localized the protein to goblet cells of intestinal metaplasia and to malignant glands—never to native gastric epithelium—suggesting that ITLN1 expression is induced as part of the CDX2-driven intestinal transdifferentiation program. Indeed, a strong positive correlation was observed between nuclear Caudal-type homeobox 2 (CDX2) and cytoplasmic omentin-1 staining (*p* < 0.001).

Clinically, ITLN1 expression was strongly associated with a favorable tumor phenotype. Among ITLN1-positive tumors, 80% exhibited intestinal-type histology compared to 61% of ITLN1-negative cases. Similarly, 88% of ITLN1-positive tumors were well or moderately differentiated (G1/G2) versus only 60% among ITLN1-negatives. Limited depth of invasion (T1/T2) was observed in 87% of ITLN1-positive cases versus 68% of those lacking ITLN1 expression. Nodal involvement (N+) occurred in just 8% of ITLN1-positive tumors, compared to 34% in the negative group, while distant metastases (M1) were present in 25% of ITLN1-positive cases, compared to 53% in ITLN1-negative patients. Functionally, ITLN1-high tumors demonstrated significantly lower cellular proliferation, with Ki-67 indices decreasing in parallel with rising ITLN1 expression (*p* = 0.001). Moreover, expression of heparinase–an extracellular matrix-degrading enzyme implicated in metastatic invasion–was inversely correlated with ITLN1 levels (*p* < 0.001). These biologic and pathologic features translated into survival outcomes: patients with ITLN1-positive tumors had a median overall survival of 44 months, substantially higher than the 25 months observed in ITLN1-negative cases. In multivariate Cox regression controlling for TNM stage, grade, and nodal status, absence of ITLN1 expression independently predicted worse prognosis, doubling the hazard of death (HR = 2.25; 95% CI: 1.23–3.98; *p* = 0.001). Collectively, the authors suggested that omentin is more than a passive marker that is induced during CDX2-mediated intestinal metaplasia as it may actively participate in suppressing tumor proliferation, invasion, and dissemination. Thus, they suggested that it might serve as a viable immunohistochemical marker and potential effector for therapeutic modulation in gastric cancer [47].

Li et al. investigated how omentin affects the behavior of gastric cancer cells by increasing ITLN1 levels in SGC-7901 and AGS gastric cancer cell lines. Forced ITLN1 expression activated a molecular chain: it silenced the PI3K → AKT → IKK axis, kept NF-κB in the cytoplasm, and thereby lifted NF-κB-mediated repression of the HNF4A promoter. Elevated Hepatocyte nuclear factor 4 alpha (HNF4α) then trapped β-catenin at the membrane, lowered TCF reporter constructs for Wnt/β-catenin signaling (TOP/FOP) luciferase output, and reduced the activity of several genes involved in cancer progression (including AXIN2, CCND2, RUNX2, and MMP3).

This molecular shift was reflected in distinct biological behaviors. Gastric cancer cells overexpressing ITLN1 formed fewer colonies, migrated more slowly in scratch assays, and generated smaller subcutaneous tumors in mice, along with a 70% reduction in lung metastases. In contrast, ITLN1-deficient cells showed the opposite behavior, which was reversed when HNF4α was reintroduced, confirming a functional link. In clinical samples, both ITLN1 and HNF4α were upregulated in 90 primary gastric tumors compared to adjacent normal mucosa. However, their expression declined significantly in tumors with poor differentiation, deeper invasion, lymph node involvement, and advanced TNM stage. The expression levels of ITLN1 and HNF4α showed strong correlation (R = 0.82), and high expression of either gene was associated with significantly improved overall survival (*p* < 0.001). Based on these findings, the authors suggested a tumor-suppressive role for omentin-1 in gastric cancer, acting through inhibition of PI3K/NF-κB signaling, restoration of HNF4α, and suppression of β-catenin activity. Loss of ITLN1 was correlated to a more aggressive tumor phenotype and worse prognosis, suggesting its relevance as both a prognostic marker and therapeutic target [48].

### 5.2. Hepato-Pancreato-Biliary (HPB) Cancer

#### 5.2.1. Hepatocellular Cancer

Emerging data suggest that omentin-1 plays a tumor-suppressive role in hepatocellular carcinoma (HCC), supported by mechanistic insights, histopathological findings, and clinical observations.

Zhang et al. demonstrated that omentin-1 exerts pro-apoptotic effects in HCC cell lines through a Sirt1-dependent p53 acetylation pathway. In vitro, exogenous omentin-1 at concentrations of 1–2 µg/mL significantly reduced proliferation and increased apoptosis in HepG2 and HuH-7 cells. Omentin-1 enhanced acetylation of p53 without altering its transcript levels, suggesting stabilization through post-translational modification. Silencing Sirt1 reversed these effects, identifying it as the primary deacetylase inhibited by omentin-1. Downstream, omentin-1 triggered mitochondrial apoptotic signaling while also activating the JNK pathway selectively. Together, these findings support a model in which omentin-1, derived from visceral fat, augments hepatocyte apoptosis via a Sirt1–p53–JNK axis, linking adipokine signaling to anti-tumor mechanisms in the liver. This pathway suggests a potential therapeutic target that links metabolic signals from visceral fat to tumor suppression in the liver [49].

Li et al. studied a cohort of 149 patients undergoing surgical resection for HCC. Quantitative analyses revealed that ITLN1 expression was significantly reduced in 78% of tumors compared to matched non-neoplastic liver tissue. Low ITLN1 levels correlated with larger tumor size (≥5 cm), poor differentiation, capsular invasion, cirrhosis, and vascular infiltration, all hallmarks of biologically aggressive disease. Notably, recurrence rates after resection were nearly four times higher in ITLN1-low tumors than in ITLN1-high ones. Survival analyses further emphasized the prognostic impact: patients with high ITLN1 expression exhibited a 5-year overall survival of 56% and disease-free survival of 36%, compared to 32% and 21%, respectively, in the ITLN1-low group. In multivariate Cox regression adjusting for tumor burden, fibrosis, and vascular features, high ITLN1 remained an independent predictor of improved outcome, halving the hazard of death and recurrence (HR ≈ 0.5). Although the mechanistic pathways were not explored in this study, the authors referenced ITLN1’s known roles in modulating HNF4α and β-catenin signaling as possible avenues for its anti-tumor function. They concluded that diminished ITLN-1 is a hallmark of biologically aggressive HCC and that restoring or mimicking its activity might offer a novel therapeutic target [50].

Furthermore, Cidem et al. identified ITLN1 as a functional receptor for bovine lactoferrin (LF), mediating its internalization and anti-tumor actions in liver cancer. Using HepG2, Hep3B, and SK-Hep1 cells, the authors demonstrated that LF binds to cell-surface ITLN1 with high affinity, triggering endocytosis and initiating downstream anti-cancer programs. LF-INTL1 engagement activated p38 MAPK and JNK pathways, leading to mitochondrial apoptosis, as well as ERK-mediated p53 upregulation and G1-phase cell cycle. Moreover, silencing ITLN1 abolished LF uptake and downstream signaling, underscoring the importance of ITLN1 in these processes. In an orthotopic nude-mouse model, oral LF (100–200 mg/kg/day) prevented hepatic tumor outgrowth, preserved body weight, and suppressed pro-angiogenic (VEGF/CD31) and cell-cycle markers (CDK2/4). Histological analysis confirmed near-complete absence of tumor foci in LF-treated livers. Collectively, the authors proposed ITLN1 as a dual-purpose molecule: (i) a predictive marker of LF sensitivity and (ii) a therapeutic target through which ligand-based or mimetic therapies might be developed to enhance apoptosis, cell-cycle control, and angiogenesis suppression in HCC [51].

#### 5.2.2. Pancreatic Cancer

The potential role of omentin-1 in pancreatic adenocarcinoma (PA) has been explored primarily through serum-based studies, with an emphasis on its diagnostic and prognostic implications. In a prospective case-control study, Karabulut et al. measured circulating omentin-1 levels in 33 treatment-naïve patients with histologically confirmed PA and compared them to 30 age-, sex-, and BMI-matched healthy controls. Serum omentin-1 concentrations were markedly elevated in the cancer cohort, with a median of approximately 9.6 ng/mL versus 1.6 ng/mL in controls—an approximate eight-fold increase (*p* < 0.001). This elevation did not correlate with demographic factors, systemic inflammation, or conventional tumor markers (CA19-9, CEA), but was significantly higher in patients with large primary tumors (≥4 cm, *p* = 0.03), suggesting a link between omentin-1 and tumor burden.

Despite its strong diagnostic separation, omentin-1 did not demonstrate prognostic value. Overall survival in the cohort was 41 weeks, and neither Kaplan–Meier nor multivariate Cox regression analyses identified omentin-1 as a significant predictor of outcome. Instead, prognosis was determined by classical factors such as age > 60 years, ECOG performance status, presence of liver metastases, and response to gemcitabine-based chemotherapy. Based on these results, the authors proposed omentin-1 as a promising adjunct diagnostic biomarker for PA, but not as a reliable predictor of prognosis or treatment response [52].

Further supporting its potential diagnostic relevance, a study by Głuszek et al. compared serum omentin-1 levels across 25 patients with pancreatic ductal adenocarcinoma (PDAC), 10 with chronic pancreatitis (CP), and 36 healthy individuals. Omentin-1 was significantly elevated in PDAC cases (mean ~582 ng/mL) compared to controls (~462 ng/mL; *p* < 0.05), while patients with CP showed intermediate levels without statistically significant differences from either group. Within the PDAC cohort, omentin-1 levels did not correlate with TNM stage, resectability, age, or BMI. Receiver operating characteristic (ROC) analysis yielded an area under the curve (AUC) of 0.73, with 65% sensitivity and 78% specificity, suggesting moderate diagnostic accuracy. Despite this, no association with clinical outcome was found [53].

### 5.3. Colorectal Cancer

The EPIC-Potsdam case-cohort analysis using baseline plasma from a random sub-cohort of 2295 adults and 251 incident cases accrued over 10.4 years provided prospective evidence that circulating omentin-1 is positively—rather than inversely—associated with colorectal cancer development. Notably, a monotonic dose-response relationship was observed: participants in the highest omentin quartile (median ≈ 563 ng/mL) had a 2.3-fold higher CRC risk than those in the lowest quartile (≈287 ng/mL), and each doubling of omentin-1 was associated with nearly a two-fold increase in relative risk (RR ≈ 1.98).

Subgroup analyses revealed a significant interaction with obesity status. In non-obese individuals (BMI < 30 kg/m^2^), the omentin–CRC association was pronounced (RR per doubling ≈ 2.3), whereas in obese individuals (BMI ≥ 30 kg/m^2^), no significant relationship was found (RR ≈ 1.1). These findings suggest that omentin-1 may act via distinct biological mechanisms depending on adiposity, possibly through differential activation of PI3K-Akt signaling, genomic instability, or immune surveillance in lean versus obese tissue microenvironments. Despite its positive correlations with protective factors such as HDL-C and adiponectin, omentin-1 did not confer cancer protection, highlighting a potential dual or context-dependent role. Overall, the author team positioned omentin-1 as a metabolically modulated biomarker that independently enhances colorectal cancer risk stratification, particularly among lean individuals, and underscored the need for mechanistic studies to unravel its paradoxical tumor-promoting effects in a non-obese context [54].

A case-control study involving 358 patients with histologically confirmed colorectal adenocarcinoma and 286 colonoscopy-verified healthy controls found that circulating omentin-1 levels are significantly elevated in CRC and reflected tumor invasiveness. Mean plasma omentin-1 was approximately double in cancer patients compared to controls (≈67 ng/mL vs. 33 ng/mL, *p* = 0.005). Stratification by tertiles revealed that individuals in the highest omentin-1 group had an adjusted 5.8-fold increased odds of CRC versus those in the lowest tertile, even after controlling for confounders. Diagnostic performance was high, with an area under the ROC curve of 0.884; at a threshold of 56.9 ng/mL, omentin-1 demonstrated 81% sensitivity and 70% specificity.

High circulating omentin-1 was also associated with more aggressive disease features: levels above the control-defined threshold were significantly linked to deeper tumor invasion (pT3/4) and advanced TNM stage (III–IV). However, omentin-1 showed no association with tumor size, side, grade, nodal status, or distant metastasis. Notably, in the CRC cohort, omentin-1 levels were not correlated with BMI, glucose, or lipid markers, indicating that its elevation reflects cancer biology rather than metabolic syndrome or obesity. Taken together, the authors underlined the role of omentin-1 as a robust and independent biomarker for CRC risk and progression, with potential applications in early detection and disease monitoring in clinical practice [55].

Feng et al. evaluated the diagnostic and prognostic significance of plasma omentin-1 in 319 patients undergoing curative resection for colorectal cancer, compared with 300 age- and sex-matched healthy controls. Pre-operative omentin-1 levels were markedly elevated in CRC patients (mean ≈ 69 ng/mL) relative to controls (≈38 ng/mL, *p* < 0.001), and although levels declined significantly post-operatively, they remained above baseline despite no evidence of disease on imaging. Cross-sectional analyses linked higher circulating omentin-1 to adverse pathological features: patients with TNM stage III/IV disease and nodal metastases had significantly higher levels than those with stage I/II or node-negative tumors (*p* < 0.01).

Using a plasma threshold of 50 ng/mL, multivariate Cox regression models adjusting for TNM stage, tumor grade, lymph-node status, and vascular invasion identified omentin-1 as an independent predictor of recurrence (HR ≈ 3.3, *p* < 0.001). During a median follow-up of 24 months, the cohort experienced a 30.6% rate of recurrence or metastasis and a 19.3% mortality rate. Elevated omentin-1 retained its prognostic value in fully adjusted models, predicting both recurrence (OR ≈ 3.3) and 2-year overall mortality (OR ≈ 2.1), with comparable strength to classical predictors such as nodal involvement and vascular tumor emboli. Based on these findings, omentin was suggested as dual-purpose biomarker in CRC: a state marker reflecting disease presence and biological aggressiveness, and a prognostic tool for stratifying risk of early postoperative relapse and death, potentially informing surveillance and adjuvant treatment decisions [56].

Maeda et al. focused on Transmembrane protein 207 (TMEM207), a previously uncharacterized transmembrane protein that was found to be an intracellular chaperone for omentin-1. Immunohistochemical analysis of 216 CRC specimens revealed TMEM207 expression in only 18% of tumors overall but in 64% of mucinous carcinomas, indicating a histotype-specific pattern. Notably, TMEM207-positive tumors exhibited a significantly lower incidence of lymph-node metastasis (2/38, 5.3%) compared to TMEM207-negative counterparts (54/178, 30.3%; *p* < 0.001), suggesting a potential role for TMEM207 in limiting metastatic dissemination.

Biochemical assays, including co-immunoprecipitation and in situ proximity-ligation, confirmed a physical interaction between TMEM207 and omentin-1 within the cytoplasm—likely in the endoplasmic reticulum-Golgi axis—suggesting that TMEM207 facilitates proper omentin-1 processing and secretion. Functional knockdown of TMEM207 in CRC cell lines (SW480, RCM-1) led to polyubiquitination and proteasomal degradation of omentin-1, significantly reducing extracellular omentin levels and, by inference, diminishing its tumor-suppressive availability in the microenvironment. The authors proposed a mechanistic axis, in which TMEM207 deficiency drives functional omentin-1 loss, compromising anti-metastatic signaling and facilitating nodal dissemination. These findings introduced TMEM207 as a potential therapeutic target, with future interventions aimed at restoring TMEM207 function or exogenous omentin-1 supplementation to limit metastatic progression in colorectal cancer [57].

A review by the same authors consolidated emerging evidence that omentin-1 (intelectin-1) may function as a tumor-suppressive adipokine in colorectal cancer, with its local bioavailability tightly linked to the processing chaperone TMEM207. While circulating omentin-1 is typically reduced in obesity—a pattern shared with adiponectin—the review highlights colorectal proteomic data indicating that tumor omentin-1 expression is over 40-fold higher in stage IV long-term survivors compared to patients with rapid disease progression, suggesting a favorable prognostic role. Additionally, TMEM207 positivity correlated with significantly fewer nodal metastases and, in stage III/IV mucinous tumors, conferred a substantial survival benefit, with 5-year disease-free survival approximately doubling (*p* = 0.014). Taken together, these findings suggest that preserved TMEM207-mediated omentin-1 secretion may characterize a less aggressive, mucin-rich CRC phenotype and serve as a favorable prognostic marker. Conversely, loss of this pathway may represent a molecular link between visceral obesity and more invasive colorectal carcinogenesis [58].

Zhang et al. demonstrated that colorectal tumors themselves are capable of producing omentin-1, establishing a tumor-autonomous source for this adipose-derived factor. Matched tumor and adjacent normal mucosa from 24 CRC patients revealed approximately 5-fold higher ITLN1 mRNA in the malignant tissue, accompanied by markedly increased protein levels as shown by immunohistochemistry. Notably, omentin-1 staining localized exclusively to the cytoplasm of neoplastic glandular epithelium, with no signal in adjacent non-malignant cells. Functionally, colorectal-cancer cell lines recapitulated this pattern: SW480 cells exhibited integral ITLN1 transcription and secreted progressively increasing amounts of omentin-1 into the culture medium over 48 h, despite retaining a substantial intracellular pool, implying active synthesis, storage, and secretion. These data highlighted an autocrine or paracrine regulatory loop in which tumor-derived omentin-1 may modulate the local microenvironment.

The authors proposed this tumor-driven omentin-1 production as a mechanistic explanation for the frequent elevation of circulating omentin-1 in CRC patients—despite systemic reductions in obesity—and implied a functional role beyond biomarker status. Given omentin-1’s known capacity to activate PI3K-Akt and JNK pathways, its autocrine secretion may support proliferative and anti-apoptotic signaling in CRC. Thus, targeting the synthesis or downstream signaling of tumor-derived omentin-1 could represent a novel therapeutic strategy in colorectal cancer, particularly in settings where systemic omentin suppression is absent [59].

Ji et al. isolated CD133^+^ tumor-initiating cells from the SW480 colorectal-carcinoma line and demonstrated that omentin-1 acts directly on this stem-cell compartment. Exposure to 1 or 2 µg ml^−1^ recombinant omentin-1 reduced sphere-forming cell growth and drove cell apoptosis; the effects intensified with both dose and duration (up to 48 h). Pharmacological blockade of phosphatidylinositol-3-kinase with LY294002 further depressed colony-forming ability and raised cell death and, when combined with omentin-1, produced an additive suppressive effect.

Mechanistically, omentin-1 markedly reduced phosphorylated-Akt without altering total Akt, confirming inactivation of the PI3K–Akt pathway. The synergy with LY294002 supports that this signaling axis is a primary target of omentin’s action in colorectal cancer stem-like cells. Taken together, the data demonstrated that omentin-1 is as a negative regulator of tumor-initiating capacity in CRC via PI3K–Akt suppression, suggesting that adipokine deficiency, common in visceral obesity, may diminish inhibitory signals on cancer stem-like cells and thus promote CRC progression [60].

Uyeturk et al. investigated circulating omentin-1 levels in 45 patients with stage III colon carcinoma who had undergone curative resection followed by standard FOLFOX-4 adjuvant chemotherapy. Two months after treatment completion, at a time point verified as recurrence-free via imaging and biomarker evaluation, plasma omentin-1 concentrations were significantly elevated compared to 35 matched healthy controls (median 618 pg/mL vs. 376 pg/mL; *p* < 0.001). This post-treatment rise could not be explained by classical metabolic confounders: fasting glucose, lipid profile, BMI, renal function, and hemoglobin levels were comparable between groups, and omentin-1 did not correlate with any of these parameters within the cancer cohort. These findings suggest a cancer- or treatment-related modulation of omentin-1, independent of metabolic status.

The authors proposed that this post-treatment upregulation reflects a shift in adipokine signaling induced by malignancy or its eradication, potentially reversing the suppression typically seen in visceral obesity. They outlined two potential, non-mutually exclusive interpretations: (i) omentin-1 elevation may represent a compensatory, anti-tumorigenic host response triggered by cytoreductive therapy; or (ii) conversely, it may reflect tumor–adipose cross-talk that promotes residual disease survival. Given that omentin-1 can exhibit both pro- and anti-tumoral functions depending on microenvironmental context, the precise biological role and prognostic implications of this post-treatment elevation remain to be fully elucidated [61].

A case–control study in 358 patients with colorectal cancer (CRC) and 286 age- and BMI-matched controls examined the functional missense variant rs2274907 A > T (Val109Asp) in the ITLN1 gene to assess its contribution to cancer susceptibility and adipokine regulation. The frequency of the minor A allele was similar between patients and controls (26.1% vs. 23.4%, *p* > 0.10), and the distribution of genotypes (TT 55% vs. 57%, AT 37% vs. 35%, AA 8% in both groups) showed no significant differences, remaining consistent with Hardy–Weinberg equilibrium (χ^2^ = 0.73, *p* = 0.69). Adjusted logistic regression controlling for metabolic and lifestyle confounders showed no significant association with CRC under either a dominant (AT + AA vs. TT: OR = 1.01; 95% CI: 0.73–1.41; *p* = 0.95) or recessive model (AA vs. TT + AT: OR = 0.93; 95% CI: 0.52–1.67; *p* = 0.82). Importantly, circulating omentin-1 levels remained significantly elevated in CRC patients compared to controls (median 196 vs. 158 ng/mL; *p* = 0.005) irrespective of genotype, suggesting that tumor-associated overexpression is not genetically predetermined by this single nucleotide polymorphism.

However, a gene–environment interaction was observed with respect to adiposity. While obesity alone (BMI ≥ 25 kg/m^2^) conferred an approximately two-fold increase in CRC risk compared to lean TT homozygotes (adjusted OR = 1.99; 95% CI: 1.06–6.27; *p* = 0.041), obese individuals carrying at least one A allele (AT or AA) exhibited a three-fold increase in risk (OR = 3.03; 95% CI: 1.17–8.25; *p* = 0.022). Furthermore, within the cancer cohort, AA homozygotes showed an over-representation of aggressive features: 67% had deep mural invasion (pT3–T4) versus 39% of T-allele carriers (*p* < 0.001), and 59% had advanced-stage disease (stage III–IV) compared to 34% of T-carriers (*p* = 0.019). These data indicate that while rs2274907 A > T does not independently influence CRC susceptibility, the A allele acts as a risk and aggressiveness modifier in the context of obesity. The authors highlighted this complex gene–environment interaction between adipokine-related genetic variants, obesity, and colorectal cancer biology, underscoring the need for further mechanistic studies and exploration of their potential role in personalized risk assessment models [62].

In a case–control study, Fazeli et al. investigated plasma omentin-1 levels in 39 newly diagnosed colorectal cancer patients compared to 30 age- and sex-matched healthy controls. They observed a dramatic increase in circulating omentin-1 among CRC patients, of approximately 203 ng/mL versus just 9 ng/mL in controls—a nearly 20-fold elevation (*p* < 0.0001). This substantial difference remained highly significant even after controlling for body mass index, waist-to-hip ratio, age, metabolic syndrome status, and lipid levels, indicating that the rise in omentin-1 was not attributable to obesity or metabolic dysfunction.

Within the CRC group, omentin-1 showed weak positive correlation with total and LDL cholesterol but was unrelated to glucose levels, BMI, or TNM stage. These findings suggest that omentin-1 elevation is an early and stage-independent feature of CRC biology, not simply a marker of tumor burden or body composition. The authors proposed that omentin-1 might serve as a diagnostic biomarker—and potentially a mechanistic contributor—to colorectal tumorigenesis via pathways distinct from the insulin-resistance and obesity-driven adipokine axis traditionally implicated in cancer risk [63].

Furthermore, a translational study highlighted the tumor-suppressive role of omentin-1 in colorectal cancer. In 229 primary CRC specimens, ITLN1 protein was absent or very low in 54.6% of tumors, while it was consistently present in adjacent normal tissue. Data from The Cancer Genome Atlas (TCGA) showed that patients with high ITLN1 expression had a 49% lower risk of death than those with low expression (HR = 0.51, *p* = 0.01). In a separate patient cohort, median survival dropped from 83.3 months in ITLN1-positive tumors to 64.1 months in ITLN1-negative ones (*p* = 0.001), and ITLN1 remained an independent prognostic factor after adjustment for other variables.

To investigate functionally, CRC cells were engineered to express normal amounts of omentin-1 and implanted into mice. These tumors grew significantly less—by ~50%—compared to control cells, even though in vitro cell growth and migration were barely affected. This suggested that omentin-1 works mainly by modifying the tumor microenvironment rather than directly inhibiting tumor-cell proliferation. Flow cytometry and immunohistochemistry revealed that tumors expressing omentin-1 had 58% fewer immunosuppressive myeloid-derived suppressor cells (MDSCs), 64% fewer endothelial progenitor cells (EPCs), and a two-fold increase in tumor-infiltrating natural killer (NK) cells. The remaining MDSCs were also less suppressive: they produced 40% less nitric oxide (NOS2) and 35% less reactive oxygen species, allowing T-cells to proliferate more effectively. Mechanistically, omentin-1 activated PI3K/AKT/GSK3β signaling, stabilized the antioxidant protein Nrf2, and suppressed NF-κB activity, leading to sharp drops in IL-17D (↓ 68%) and CXCL2 (↓ 72%) secretion, two cytokines that normally attract MDSCs and EPCs. Blocking the PI3K pathway reversed these effects, confirming that omentin-1 acts through this route. Collectively, these data showed that approximately half of CRCs downregulate ITLN1 expression, potentially facilitating immune evasion and neoangiogenesis. Reconstitution of omentin-1 restores a more immunogenic and less vascularized tumor microenvironment, offering a promising therapeutic strategy aimed not at tumor cells per se, but at the stromal and immune components that enable tumor progression [64].

In a proteomic study of stage-IV colorectal cancer, omentin-1 emerged as one of the strongest biomarkers distinguishing long-term survivors from those with rapid disease progression. Kim et al. analyzed tumor samples to identify proteins differentially expressed between patients with good prognosis (survival > 5 years) and those with poor prognosis (death ≤ 25 months). Out of 1044 quantified proteins, 175 showed ≥2-fold differences, and ITLN1 had the largest increase in the good-prognosis group, with a 41.4-fold rise in isotope-coded affinity tag signal. Follow-up validation confirmed these findings. Western blotting showed an 11.5-fold higher ITLN1 protein level in long-surviving tumors (*p* = 0.03), and an independent patient set reproduced this with a near-identical 11.6-fold difference (*p* < 0.05). When combined with four other top-performing proteins (FABP1, TPM2, TAGLN, and VCP) in a logistic regression model, ITLN1 helped generate a near-perfect classification (AUC = 1.00), though ITLN1 alone still yielded an AUC of 0.69, the highest individual contribution.

Histologically, strong cytoplasmic ITLN1 staining was seen in 33% of good-prognosis tumors but in none of the poor-prognosis group, aligning with the biochemical data. Functional pathway analysis placed ITLN1 among a small cluster of proteins enriched in favorable cases, contrasting with the dominant up-regulation of cytoskeletal and migration-related pathways in aggressive tumors. Altogether, based on these results, the authors identified omentin-1 as a robust prognostic discriminator in advanced CRC, with expression levels that differ ~40-fold between survival extremes. Their data suggested that preserved ITLN1 may reflect or contribute to a less invasive tumor phenotype, and supported its use as a molecular marker for outcome prediction in metastatic colorectal cancer [65].

A comprehensive overview of key clinical and preclinical studies evaluating the role of omentin-1 (ITLN1) across gastrointestinal malignancies is provided in Table 1 and Table 2, respectively.

## 6. Directions and Research Needs

### 6.1. What Is Missing About Omentin’s Role in GI Cancers?

Despite substantial advances in understanding the biological role and clinical implications of omentin in gastrointestinal cancers, several critical knowledge gaps remain, limiting its definitive application as a biomarker or therapeutic target.

In esophageal cancer, key gaps remain in understanding how omentin is altered and what functional role it plays. One priority is to gather data on circulating omentin-1 levels in esophageal adenocarcinoma (EAC) and squamous cell carcinoma (ESCC) patients, using patient cohorts and adjusting for obesity-related factors. Esophageal adenocarcinoma often arises in the context of visceral obesity, which itself lowers circulating omentin-1 levels, whereas ESCC patients are typically non-obese; thus, future studies should measure omentin-1 in EAC and ESCC while controlling for visceral adiposity and insulin resistance to detect true cancer-specific changes [43]. Another open question is the cellular distribution of ITLN1 in esophageal tissues. Applying transcriptomics or advanced immunohistochemistry along the esophageal epithelium (from normal mucosa through Barrett’s metaplasia to carcinoma) could map which cell types express ITLN1 and how this varies with disease stage [46]. A third gap is the lack of functional studies defining omentin’s role in esophageal tumor biology and how it affects tumor growth or modulates pathways like inflammation and apoptosis.

Regarding gastric cancer, future research priorities include the prospective validation of ITLN1 as a routine prognostic biomarker across diverse patient groups and treatment modalities. Additionally, serum-based studies with adequate power are needed to clarify whether circulating omentin-1 changes after curative gastrectomy or during adjuvant therapy. It is also important to investigate recombinant omentin or HNF4α activators in preclinical models, such as patient-derived gastric-cancer organoids and xenografts [47]. Finally, combination-therapy studies should test for synergy between omentin pathway activation and immune-checkpoint blockade, particularly in Microsatellite instability (MSI)-high disease.

For hepatocellular carcinoma, research efforts should focus on the prospective validation of ITLN1 as a prognostic biomarker across HBV-, HCV-, and MASLD-related HCC. Pharmacokinetic and pharmacodynamic evaluations of recombinant omentin-1 in vivo models of liver cancer are also warranted to better characterize its therapeutic potential. Further research should explore combination strategies evaluating omentin pathway activation with current first-line agents, such as atezolizumab plus bevacizumab or lenvatinib, to test for additive or synergistic effects. Moreover, large cohorts are required in order to examine its role as serum biomarker for minimal-residual disease or early recurrence post-resection [50].

In pancreatic cancer, several gaps need to be addressed. The source of markedly elevated circulating omentin-1 remains unknown, necessitating further investigation to determine whether this increase is driven by tumor-derived production, interactions with cancer-associated adipocytes, or a broader systemic inflammatory response [52]. Mechanistic studies are absent, making it crucial to investigate whether omentin-1 plays a causal role in pancreatic cancer biology using appropriate in vitro and in vivo models. The utility of serum omentin-1 as a diagnostic biomarker also requires validation in large, prospective studies to confirm its accuracy in differentiating early-stage cancer from non-malignant conditions like chronic pancreatitis [53].

In the context of colorectal cancer, a key challenge is understanding why high blood levels of omentin-1 are linked to an increased cancer risk, while high levels of omentin-1 within the tumor itself predict better patient survival. This paradox suggests a clear difference between omentin circulating in the bloodstream, which might simply indicate conditions like obesity, and omentin acting locally within tumor. Resolving this contradiction likely involves studying the molecule TMEM207, which helps omentin fold properly and function effectively. It is believed that TMEM207 ensures omentin is correctly structured and released specifically in the tumor environment, allowing it to act as an anti-cancer agent. If this is true, circulating omentin might be inactive or simply a marker, while TMEM207-regulated omentin within the tumor microenvironment may exert active anti-tumor effects. The tumor-suppressive effects of locally active omentin-1 likely involve modulation of the tumor microenvironment, specifically through the reduction of immunosuppressive myeloid-derived suppressor cells (MDSCs) and the inhibition of angiogenesis via suppression of endothelial progenitor cells (EPCs). Understanding exactly how omentin-1 prevents these cells from promoting tumor growth is vital. Additionally, omentin-1 might strongly counteract cancer stem cells and support tumor-suppressive microRNAs, which are small molecules that help regulate cancer growth. These cellular-level effects might explain why local omentin-1 is protective and could be especially beneficial for patients at higher risk, such as those with diabetes. Overall, addressing the systemic (blood-based) versus local (tumor-based) paradox of omentin requires integrating molecular (TMEM207), environmental (MDSCs/EPCs), and cellular (cancer stem cells) mechanisms [57].

It is important to note that the available literature is uneven across gastrointestinal malignancies. The majority of clinical and mechanistic studies focus on colorectal cancer, while gastric, hepatic, pancreatic, and esophageal cancers are represented by relatively few investigations. This imbalance likely reflects both the high global incidence of colorectal cancer and the availability of large prospective cohorts in which circulating omentin has been measured. However, it limits the generalizability of conclusions to other GI tumors and highlights the need for dedicated studies in less-studied cancer types.

### 6.2. Clinical Utility

Beyond its biological roles, omentin carries several potential clinical applications in gastrointestinal cancers. In colorectal cancer, elevated circulating omentin-1 has shown diagnostic accuracy comparable to conventional tumor markers, while intratumoral ITLN1 expression and its stabilization by TMEM207 may serve as independent prognostic indicators and therapeutic modulators [57]. In hepatocellular carcinoma (HCC), loss of ITLN1 correlates with aggressive clinicopathological features and poor outcomes, and restoration of omentin signaling—either directly or via lactoferrin-ITLN1 interactions—has demonstrated anti-tumor efficacy in preclinical models [49,50,51]. In gastric cancer, ITLN1 positivity associates with favorable histological features, reduced metastatic potential, and improved overall survival, underscoring its potential use as an immunohistochemical prognostic biomarker [47,48]. By contrast, in pancreatic cancer, elevated circulating omentin-1 appears more relevant diagnostically than prognostically, with studies suggesting a role as an adjunct serum marker alongside CA19-9 [52,53].

Mechanistically, these findings point to omentin as a modulator of pathways central to tumor progression, including PI3K/NF-κB, HNF4α/β-catenin, and Sirt1–p53–JNK signaling, as well as its regulation via TMEM207-mediated stabilization. This highlights its dual value as both a biomarker (diagnostic and prognostic) and a therapeutic co-factor, raising the possibility of targeting this axis to restore tumor-suppressive activity. These clinical perspectives highlight the need for validation in larger, multi-omics, and prospective studies, as discussed in the following section.

### 6.3. Proposals for Future Research Focusing on Translational and Clinical Studies

To effectively translate the current understanding of omentin into clinical practice, future research must focus on validating its biomarker potential, clarifying unresolved biological inconsistencies, and investigating its viability as a therapeutic target.

A key translational priority is validating omentin as a robust clinical biomarker through large-scale, prospective clinical studies designed to assess its prognostic and diagnostic value. For instance, prospective cohort trials examining ITLN1 expression in gastric cancer, hepatocellular carcinoma, and colorectal cancer using standardized immunohistochemical protocols are essential for ensuring reproducible and clinically meaningful results. Another priority is to investigate depot-specific expression of omentin in cancer, since visceral adipose tissue is the primary source of omentin, whereas subcutaneous adipose tissue expresses it at much lower levels. Whether differential expression between VAT and subcutaneous fat contributes to cancer development, progression, or cachexia remains unknown, and future studies should address this as a potential determinant of systemic versus tumor-localized omentin activity. Similarly, in malignancies like pancreatic cancer, where elevated circulating omentin-1 levels indicate possible diagnostic utility, carefully designed case-control studies are needed to assess its sensitivity and specificity, particularly when combined with established biomarkers such as CA19-9. Standardizing assay methodologies, including Enzyme-linked immunosorbent assay (ELISA) protocols for circulating omentin-1 quantification, is essential to ensure data consistency, reproducibility, and comparability across different studies. Importantly, future investigations should also elucidate the relationship between omentin-1 expression and surgical outcomes, including the impact of different resection strategies on circulating and tissue omentin-1 levels. Understanding whether surgical cytoreduction modifies omentin signaling, either through tumor removal or changes in systemic inflammation, may provide critical insights into its role as a dynamic biomarker.

Moreover, addressing existing biological paradoxes associated with omentin-1 remains a critical obstacle in clinical translation. A prominent challenge is the contradictory evidence surrounding omentin-1 in CRC, wherein elevated circulating levels appear associated with increased risk, while increased tumor-localized expression correlates with protective effects. Comprehensive cohort studies that concurrently measure circulating omentin-1, tumor-localized ITLN1 expression, its chaperone protein TMEM207, and detailed patient metabolic profiles are thus needed. These integrated analyses can elucidate the mechanistic distinctions underlying systemic versus localized omentin dynamics. Moreover, the underlying origin of increased circulating omentin-1 in pancreatic and colorectal cancers should be explored using biologically relevant in vitro and ex vivo models that simulate tumor–host interactions. Advanced single-cell RNA sequencing should be employed to map ITLN1 expression and its receptors within the tumor microenvironment, clarifying the cellular origin and targets of omentin-1 signaling across gastrointestinal malignancies.

Lastly, translational efforts prioritize the systematic investigation of omentin as a therapeutic agent. Given its established metabolic and immune-regulatory role, future research should explore potential synergistic effects between omentin signaling enhancement and existing therapeutic approaches, such as metabolic modulators (e.g., metformin) or immune checkpoint inhibitors (e.g., PD-1 blockade). In malignancies where omentin-1 demonstrates tumor-suppressive activity—such as gastric cancer, hepatocellular carcinoma, and specific subtypes of colorectal cancer—direct therapeutic approaches using recombinant omentin-1 or newly developed omentin-mimetic compounds should be evaluated in preclinical models. Additionally, targeting the TMEM207–omentin-1 signaling axis represents an innovative therapeutic strategy. Moreover, the TMEM207–omentin-1 signaling pathway offers a novel therapeutic target. Developing small molecules or biologics that enhance or stabilize the TMEM207–omentin interaction may promote endogenous production of functional omentin-1, thereby enhancing its anti-metastatic and tumor-suppressive effects. Such targeted therapeutic strategies hold substantial promise for personalized therapeutic approaches in gastrointestinal oncology.

## 7. Conclusions

Omentin links metabolic disorders with gastrointestinal cancers, displaying a paradoxical dual role: tumor-suppressive locally yet systemically elevated in association with cancer risk. Current evidence supports its potential as a diagnostic and prognostic biomarker, as well as a therapeutic target, though validation is still lacking. Future studies should focus on large prospective cohorts and multi-omics approaches to clarify systemic versus local effects and to establish its clinical utility. Exploration of omentin-based therapies, including recombinant proteins or pathway modulators, may promote its integration into clinical practice in gastrointestinal oncology.

## Figures and Tables

**Table 1:** Structured summary of clinical studies investigating omentin in gastrointestinal cancers including experimental model used, key findings, proposed biological role, and relevant clinical implications. Abbreviations: CRC: Colorectal cancer; ESCC: Esophageal squamous cell carcinoma; EAC: Esophageal adenocarcinoma; BE: Barrett’s esophagus; ITLN1: Intelectin-1; OS: Overall survival; RFS: Recurrence-free survival; qRT-PCR: Quantitative real-time polymerase chain reaction; IHC: Immunohistochemistry; ELISA: Enzyme-linked immunosorbent assay; PA: Pancreatic adenocarcinoma; PDAC: Pancreatic ductal adenocarcinoma; CP: Chronic pancreatitis; AUC: Area under the curve; HR: Hazard ratio; SNP: Single-nucleotide polymorphism; TCGA: The Cancer Genome Atlas; WB: Western blot; EPC: Endothelial progenitor cell; MDSC: Myeloid-derived suppressor cell.

Authors and Year	Cancer Type	Model	Findings	Role	Clinical Implication
Zhang et al., 2020 [59]	ESCC	ESCC (microarray + qRT-PCR)	↑ ITLN1 linked to better OS/RFS; protective gene in 6-gene immune signature	Protective, prognostic	Favorable immune profile and improved survival
Liang et al., 2021 [45]	ESCC	ESCC tumor and adjacent normal tissue	↓ ITLN1 in tumors; associated with better immune contexture and lower immune risk score	Immune-modulating, prognostic	Immune biomarker; favorable prognosis
Busslinger et al., 2021 [46]	EAC	Barrett’s esophagus and dysplasia/EAC tissue	ITLN1 expressed in BE epithelium; unchanged in progression to dysplasia or adenocarcinoma	Marker of metaplasia	Diagnostic utility in Barrett’s; no role in malignant transformation
Zheng et al., 2012 [47]	Gastric	qPCR, IHC, survival analysis on tumors and normal tissue	ITLN1 mRNA 8× higher in tumors; associated with CDX2 expression, favorable histology, lower stage, improved survival (HR = 2.25 for ITLN1−)	Tumor suppressor downstream of CDX2	Prognostic marker and therapeutic target for intestinal-type GC
Li et al., 2023 [50]	hCC	149 HCC patients (tissue analysis)	↓ ITLN1 in 78% of tumors; associated with aggressive features and worse survival (HR ≈ 0.5)	Prognostic biomarker	Risk stratification tool post-resection
Karabulut et al., 2016 [52]	Pancreatic	33 PA patients vs. 30 controls (ELISA)	↑ Omentin-1 (~9.6 vs. ~1.6 ng/mL); correlates with tumor size, not OS or chemo response	Diagnostic marker	Potential biomarker for detection, not for prognosis
Kiczmer et al., 2018 [53]	Pancreatic	25 PDAC, 10 CP, 36 controls (serum)	↑ Omentin-1 in PDAC vs. controls; AUC 0.73; no correlation with TNM stage or BMI	Diagnostic adjunct	Support diagnosis; limited prognostic use
Aleksandrova et al., 2016 [54]	CRC	Prospective cohort (n = 2295 subcohort + 251 CRC cases)	High baseline omentin-1 associated with increased CRC risk, especially in non-obese	Risk biomarker	Stratify risk, particularly in lean individuals
Zhao et al., 2019 [55]	CRC	Case-control (358 CRC vs. 286 controls)	↑ Omentin-1 in CRC; associated with advanced stage (pT3/4, TNM III–IV); AUC 0.88	Diagnostic and staging marker	Non-invasive diagnostic tool with good sensitivity
Feng et al., 2020 [56]	CRC	Prospective cohort (319 CRC vs. 300 controls)	Elevated pre-op omentin-1 predicts recurrence (HR 3.3) and mortality (HR 2.1)	Prognostic biomarker	Post-operative monitoring and treatment
Uyeturk et al., 2014 [61]	CRC	Stage III post-FOLFOX patients (n = 45)	↑ Omentin-1 after chemo; not correlated with metabolic parameters	Reactive or compensatory marker	Host response to therapy
Zhang et al., 2021 [62]	CRC	Genetic association study (SNP)	Variant not associated with CRC overall; modifies risk in obesity	Gene–environment interaction	Stratify risk in obese patients based on genotype
Fazeli et al., 2013 [63]	CRC	39 CRC vs. 30 controls	≈20× higher plasma omentin-1 in CRC; independent of BMI or stage	Carcinogenesis biomarker	Early detection
Chen et al., 2021 [64]	CRC	TCGA + IHC (n = 229)	Low ITLN1 linked to worse OS; re-expression reduces EPCs and MDSCs	Microenvironment modulator	Therapeutic restoration enhances anti-tumor immunity
Kim et al., 2012 [65]	CRC	Stage IV CRC proteomics	ITLN1 41× higher in long-term survivors; validated by WB/IHC	Prognostic biomarker	Survival indicator; guides treatment intensity

**Table 2:** Structured summary of preclinical and mechanistic studies investigating omentin in gastrointestinal cancers including experimental models, molecular findings, proposed biological role, and translational implications. Abbreviations: CRC: Colorectal cancer; HCC: Hepatocellular carcinoma; ITLN1: Intelectin-1; OS: Overall survival; IHC: Immunohistochemistry; siRNA: Small interfering RNA; PI3K: Phosphoinositide 3-kinase; NF-κB: Nuclear factor kappa-light-chain-enhancer of activated B cells; HNF4α: Hepatocyte nuclear factor 4 alpha; SIRT1: Sirtuin 1; JNK: c-Jun N-terminal kinase; LF: Lactoferrin; CD133^+^: Cancer stem cell marker; Akt: Protein kinase B.

Authors and Year	Cancer Type	Model	Findings	Role	Clinical Implication
Li et al., 2015 [49]	Gastric	In vitro (SGC-7901, AGS), in vivo (xenografts)	ITLN1 inhibits PI3K/NF-κB, restores HNF4α, suppresses β-catenin; reduces invasion/metastasis; positively correlates with OS (R = 0.82)	Tumor-suppressive adipokine	Dual biomarker with HNF4α; therapeutic target
Zhang et al., 2013 [50]	HCC	HepG2, HuH-7 cell lines	Omentin-1 stabilizes p53 via SIRT1 inhibition; activates caspase-mediated apoptosis via JNK	Tumor suppressor	Potential therapeutic via p53 reactivation
Cidem et al., 2024 [52]	HCC	HepG2, Hep3B, SK-Hep1 + orthotopic mice	LF requires omentin-1 for uptake; triggers apoptosis, cell-cycle arrest, inhibits angiogenesis	Therapeutic co-factor	Predictive biomarker and therapeutic enhancer for LF-based treatment
Maeda et al., 2016 [58]	CRC	IHC + in vitro (siRNA)	TMEM207 loss reduces omentin secretion; correlates with nodal metastasis	Post-translational regulator	Potential target for therapy and prognosis
Zhang et al., 2020 [60]	CRC	CRC tissue + SW480 cells	Tumors overexpress omentin-1; secreted in autocrine/paracrine fashion	Context-dependent role	Targeting tumor-derived omentin alters CRC progression
Ji et al., 2019 [61]	CRC	CD133^+^ SW480 cells	Omentin-1 inhibits PI3K/Akt; reduces stemness and promotes apoptosis	Cancer stem cell suppressor	Adjuvant therapy targeting cancer stem cells

## Data Availability

Data supporting the findings of this study are available from the corresponding author upon reasonable request.

## Supplementary Materials

The following supporting information can be downloaded at: https://www.mdpi.com/article/10.3390/metabo15100649/s1, Figure S1: Trial flow of this review.

## Abbreviations

The following abbreviations are used in this manuscript:

Akt	Protein kinase B
AMPK	AMP-activated protein kinase
AUC	Area under the curve
BE	Barrett’s esophagus
CA19-9	Carbohydrate antigen 19-9
CEA	Carcinoembryonic antigen
CDX2	Caudal-type homeobox 2
CP	Chronic pancreatitis
CRC	Colorectal cancer
CSC	Cancer stem cell
CXCL2	C-X-C motif chemokine ligand 2
EAC	Esophageal adenocarcinoma
EPC	Endothelial progenitor cell
ERK	Extracellular signal–regulated kinase
ESCC	Esophageal squamous cell carcinoma
HCC	Hepatocellular carcinoma
HNF4α	Hepatocyte nuclear factor 4 alpha
HOMA-IR	Homeostatic model assessment of insulin resistance
HR	Hazard ratio
IHC	Immunohistochemistry
ITLN1	Intelectin-1 (gene encoding omentin-1)
JNK	c-Jun N-terminal kinase
LF	Lactoferrin
MAPK	Mitogen-activated protein kinase
MASLD	Metabolic-associated steatotic liver disease
MASH	Metabolic-associated steatohepatitis
MDSC	Myeloid-derived suppressor cell
miRNA	MicroRNA
MSI	Microsatellite instability
NF-κB	Nuclear factor kappa B
NK cell	Natural killer cell
Nrf2	Nuclear factor erythroid 2–related factor 2
OR	Odds ratio
PA	Pancreatic adenocarcinoma
PDAC	Pancreatic ductal adenocarcinoma
PI3K	Phosphoinositide 3-kinase
PPAR-δ	Peroxisome proliferator-activated receptor delta
qRT-PCR	Quantitative real-time polymerase chain reaction
RFS	Recurrence-free survival
ROC	Receiver operating characteristic
ROS	Reactive oxygen species
SIRT1	Sirtuin 1
SNP	Single-nucleotide polymorphism
TCGA	The Cancer Genome Atlas
TMEM207	Transmembrane protein 207
TNF-α	Tumor necrosis factor alpha
TNM	Tumor–node–metastasis (staging)
TOP/FOP	TCF reporter constructs for Wnt/β-catenin signaling
VAT	Visceral adipose tissue
VCAM-1	Vascular cell adhesion molecule 1
VEGF	Vascular endothelial growth factor
VLDL	Very low–density lipoprotein
