# Supplementary figures and images for: The Role of Omentin in Gastrointestinal Cancer: Diagnostic, Prognostic, and Therapeutic Perspectives
Source: Metabolites. 2025 Sep 30;15(10):649. doi: 10.3390/metabo15100649 (PMC12566161; doi:10.3390/metabo15100649)

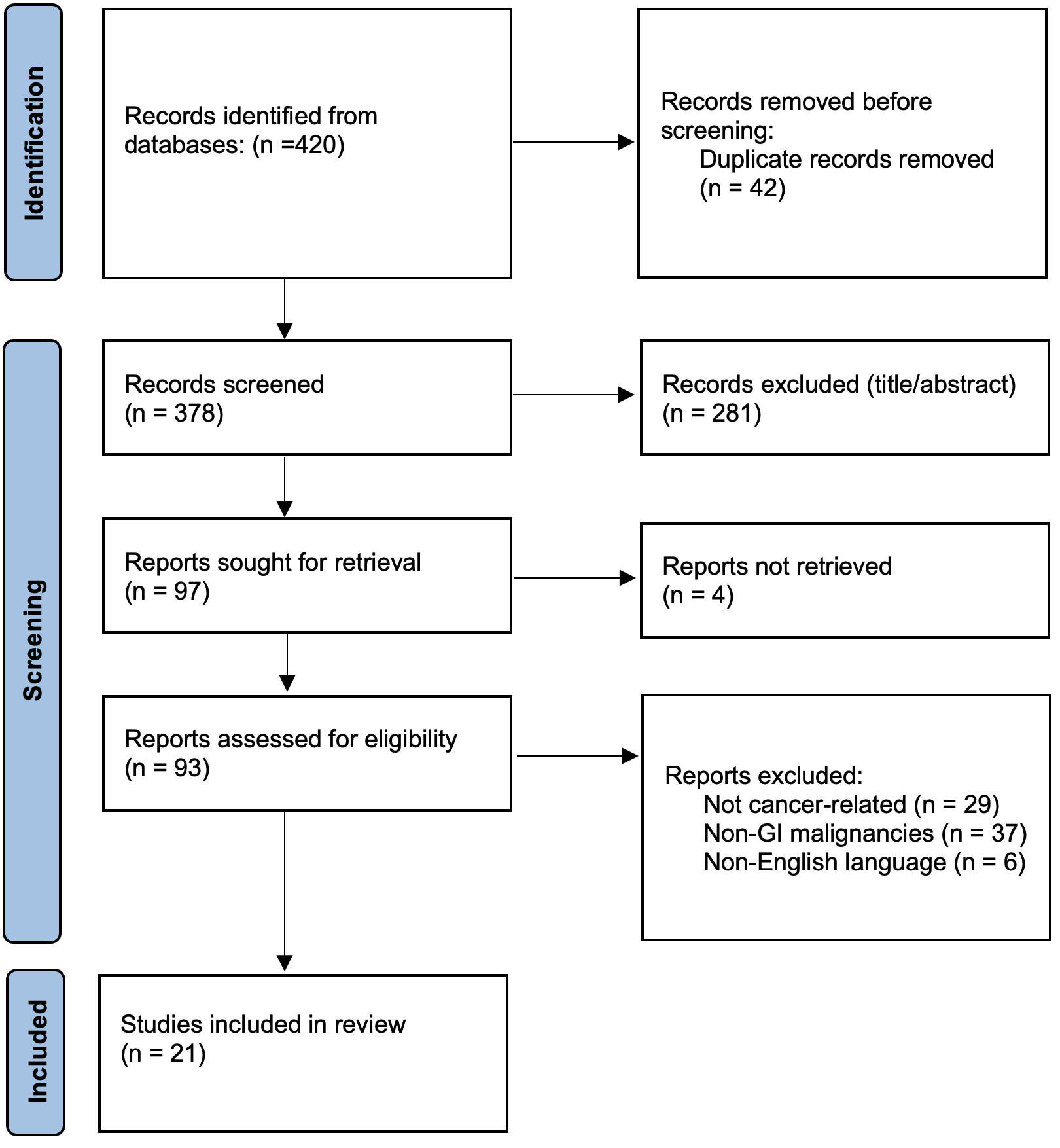

Supplement: Supplementary file 1 [file metabolites-15-00649-s001.zip › metabolites-3879198-supplementary.png]
